# Harmful Iron-Calcium Relationship in Pantothenate kinase Associated Neurodegeneration

**DOI:** 10.3390/ijms21103664

**Published:** 2020-05-22

**Authors:** Paolo Santambrogio, Maddalena Ripamonti, Chiara Paolizzi, Celeste Panteghini, Miryam Carecchio, Luisa Chiapparini, Marzia Raimondi, Alicia Rubio, Ivano Di Meo, Anna Cozzi, Stefano Taverna, Giuseppe De Palma, Valeria Tiranti, Sonia Levi

**Affiliations:** 1 IRCCS San Raffaele Scientific Institute, 20132 Milan, Italy; santambrogio.paolo@hsr.it (P.S.); cozzi.anna@hsr.it (A.C.); taverna.stefano@hsr.it (S.T.); 2 Division of Neuroscience, Vita-Salute San Raffaele University, 20132 Milan, Italy; ripamonti.maddalena@hsr.it (M.R.); chiarapaolizzi@gmail.com (C.P.); marziaraimondi@gmail.com (M.R.); 3 Medical Genetics and Neurogenetics Unit, Fondazione IRCCS Istituto Neurologico C. Besta, 20126 Milan, Italy; celeste.panteghini@istituto-besta.it (C.P.); ivano.dimeo@istituto-besta.it (I.D.M.); valeria.tiranti@istituto-besta.it (V.T.); 4 Department of Neuroscience, University of Padua, 35122 Padua, Italy; miryam.carecchio@unipd.it; 5 Neuroradiology Unit, Fondazione IRCCS Istituto Neurologico C. Besta, 20133 Milan, Italy; luisa.chiapparini@istituto-besta.it; 6 Institute of Neuroscience, National Research Council, 20132 Milan, Italy; rubiogarrido.alicia@hsr.it; 7 Department of Medical and Surgical Specialities, University of Brescia, 25123 Brescia, Italy; giuseppe.depalma@unibs.it

**Keywords:** neurodegeneration, PKAN (pantothenate kinase-associated neurodegeneration), NBIA (neurodegeneration with brain iron accumulation), iron, calcium, iPSC (induced pluripotent stem cells)

## Abstract

Pantothenate Kinase-associated Neurodegeneration (PKAN) belongs to a wide spectrum of diseases characterized by brain iron accumulation and extrapyramidal motor signs. PKAN is caused by mutations in PANK2, encoding the mitochondrial pantothenate kinase 2, which is the first enzyme of the biosynthesis of Coenzyme A. We established and characterized glutamatergic neurons starting from previously developed PKAN Induced Pluripotent Stem Cells (iPSCs). Results obtained by inductively coupled plasma mass spectrometry indicated a higher amount of total cellular iron in PKAN glutamatergic neurons with respect to controls. PKAN glutamatergic neurons, analyzed by electron microscopy, exhibited electron dense aggregates in mitochondria that were identified as granules containing calcium phosphate. Calcium homeostasis resulted compromised in neurons, as verified by monitoring the activity of calcium-dependent enzyme calpain1, calcium imaging and voltage dependent calcium currents. Notably, the presence of calcification in the internal *globus pallidus* was confirmed in seven out of 15 genetically defined PKAN patients for whom brain CT scan was available. Moreover, we observed a higher prevalence of brain calcification in females. Our data prove that high amount of iron coexists with an impairment of cytosolic calcium in PKAN glutamatergic neurons, indicating both, iron and calcium dys-homeostasis, as actors in pathogenesis of the disease.

## 1. Introduction

Pantothenate Kinase-associated Neurodegeneration (PKAN, OMIM #234200) belongs to the heterogeneous group of neurodegenerative diseases known as Neurodegeneration with Brain Iron Accumulation (NBIA) [1,2], characterized by a massive iron accumulation in the basal ganglia, progressive dystonia and parkinsonism, cognitive decline and psychiatric disturbances. PKAN represents the most common form of NBIA, covering about 50% of cases. The disease may present with a different phenotype, depending on the age of onset: in the classical form, the onset occurs in the first decade of life; in the atypical form, the onset is delayed, occurring at around the second or third decade [3,4,5]. In the classical form, patients have difficulty walking and postural symptoms associated with severe progressive dystonia, dysarthria, choreoathetosis, spasticity, hyperreflexia and retinopathy. The atypical form, on the other hand, is characterized by a combination of dystonia and parkinsonism with slower disease progression, pyramidal signs, cognitive decline and psychiatric symptoms. Disease diagnosis is carried out with brain MR T2-weighted or magnetic susceptibility (T2 *, SWI) images, capable of identifying iron accumulation frequently associated with the characteristic ‘eye-of-the-tiger’ sign. It consists of atrophy and T2 hypointensity of bilateral *globus pallidus* associated with a small hyperintensity in its anterior and medial part; signal drop is due to iron accumulation and hyperintense portion is caused by gliosis and spongiosis [3]. PKAN is an autosomal recessive rare disease caused by mutations in the *PANK2* gene, located on chromosome 20p13. Mutations in the gene are mainly missense but cases of duplication, deletions, mutations affecting splicing sites and exon deletions [6] have also been reported. The *PANK2* gene is expressed in almost all tissues with higher levels in the liver and brain [7] and encodes the PANK2 enzyme that catalyzes the first biosynthesis reaction of Coenzyme A (CoA): the phosphorylation of pantothenate (vitamin B5) in 4’-phosphopanthothenate. The PANK2 protein represents one of the four isoforms present in the human genome and localizes in the mitochondrial intermembrane space [8], while the other PANK proteins (PANK1a and b, PANK3 and PANK4) are found in the cytosol [9,10].

To date, only symptomatic treatments aimed at reducing dystonia, parkinsonism and spasticity are available without any influence on disease progression [4]. The first evidence of a decrease in disease progression in a consistent number of patients, comes from an 18-month, randomized, double-blind, placebo-, controlled trial (TIRCON2012V1) in which 49 patients were treated with the iron-chelator deferiprone [11]. Another promising approach, at least in animal models, is to treat congenital defects in CoA production by the administration of the molecule itself or its precursors [12,13,14,15]. Recently, a treatment with fosmetpantothenate, a phosphopanthothenate derivative, on a single patient with atypical PKAN, demonstrated an improvement of all clinical parameters evaluated [16]. However, despite the intensive studies conducted on cellular and animal models, together with underway clinical trials, the knowledge of the pathogenetic mechanisms that lead to PKAN disease is still in its infancy, and further studies are in progress to identify an effective therapy [15,16,17,18,19].

We previously obtained human Induced Pluripotent Stem Cells (iPSCs) by reprogramming fibroblasts of three PKAN patients and three healthy controls [18]. We differentiated iPSCs into glutamatergic neurons (iPS-derived neurons), which showed aberrant mitochondria characterized by profound structural and functional alterations, including deficiency in two iron-dependent mitochondrial biosynthetic pathways: Iron Sulphur Cluster (ISC) and haem. PKAN iPS-derived neurons also showed an increase in ROS production and a reduction in the amount of reduced glutathione, indicating an increase in oxidative stress. These defects resulted in an inability to sustain repetitive action potential firing in response to the injection of depolarizing currents [18]. Furthermore, in this study, the efficacy of CoA administration to revert the pathological phenotype in PKAN iPS-derived neurons was demonstrated. The molecule was able to improve the electrophysiological properties of the iPS-derived neurons of affected patients, to inhibit their death, to prevent the formation of ROS and to recover haem biosynthesis and respiratory activity [18].

In this work, we performed a further characterization of PKAN iPS-derived neurons, highlighting the higher amount of total cellular iron, mitochondrial calcium accumulation, an enhanced content of cytosolic calcium and a profound alteration of its homeostasis. Most importantly, calcium overload was confirmed in the brain of some PKAN patients, who underwent CT scan, showing the presence of calcifications in the medial *globus pallidus* with the prevalence in female with respect to male. These data point to an iron-calcium interplay in the pathogenesis of the disease.

## 2. Results

### 2.1. PKAN iPS-Derived Neurons Show Altered Iron Content and Deposit of Calcium Phosphate in Mitochondria

**Figure 1:**
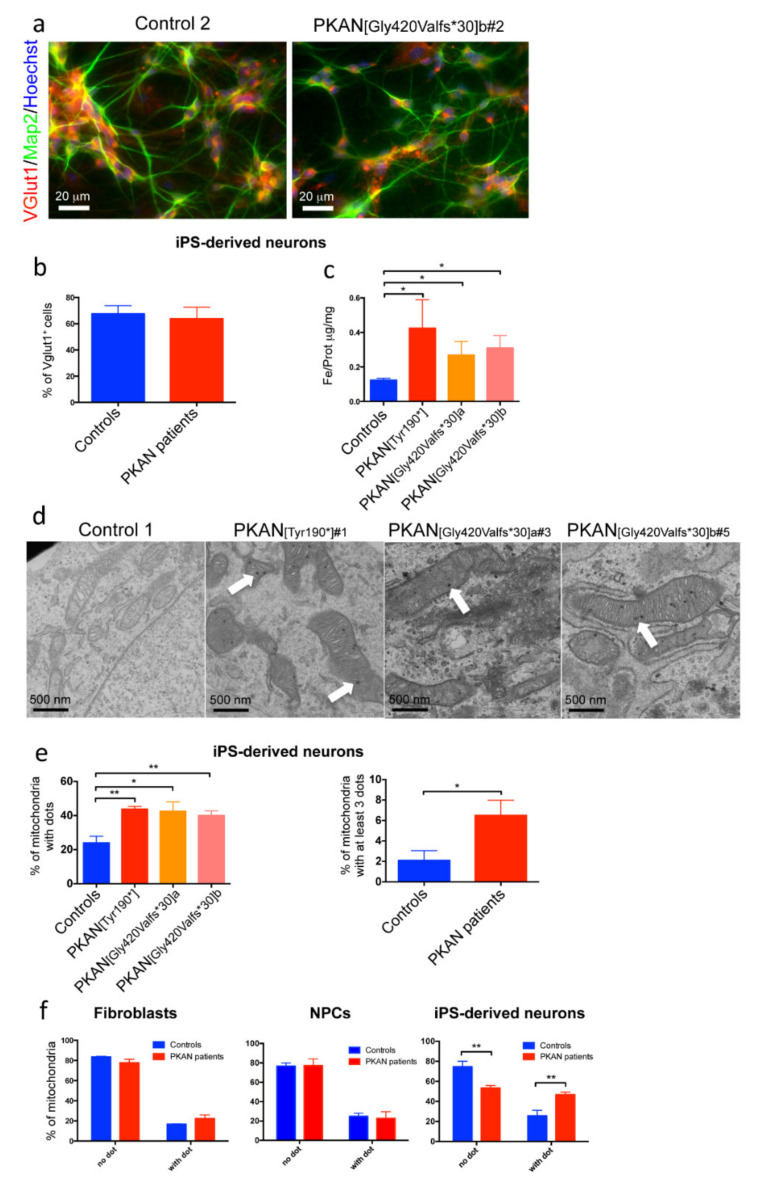
Characterization of iPS-derived neurons. (**a**) An example of iPS-derived neurons from control and one Pantothenate Kinase-associated Neurodegeneration (PKAN) patient. They were stained for neuronal markers: microtubule associated protein 2 (Map2), the vesicular glutamate transporter 1 (VGlut1) and the nuclear stain Hoechst. Scale bar 20 µm. (**b**) Plot representing the percentage of the VGlut1 positive cells. (**c**) Iron quantification by inductively coupled plasma mass spectrometry in cell lysates. * *p* < 0.05 (Kruskal-Wallis test). (**d**) Representative images of ultrastructural analysis of fixed neurons examined with electron microscope. Scale bar 500 nm. Arrows point to electron dense granules present in mitochondria. (**e**) Left panel, mitochondrial aggregates were counted in each mitochondria in >30 field (200 mitochondria total) for each sample. * *p* < 0.05; ** *p* < 0.01, (one-way ANOVA). Right panel, plot of the mitochondria with at least 3 dots. * *p* < 0.05 (Student’s *t*-test). (**f**) Percentage of mitochondria with or without dots in fibroblasts, in Neuronal Precursor Cells (NPCs) and iPS-derived neurons. ** *p* < 0.01 (two-way Anova). All data are presented as mean + SEM on at least three independent replicates.

The human PKAN neuronal model was obtained by differentiation of neuronal precursor cells (NPC) transduced with neurogenin-2 (Ngn2), as previously described [18]. After three weeks of differentiation, the iPS-derived neurons were analyzed in immunofluorescence with two neuronal markers: microtubule-associated protein 2 (Map2) and vesicular glutamate transporter 1 (Vglut1) (Figure 1a).

**Figure 2:**
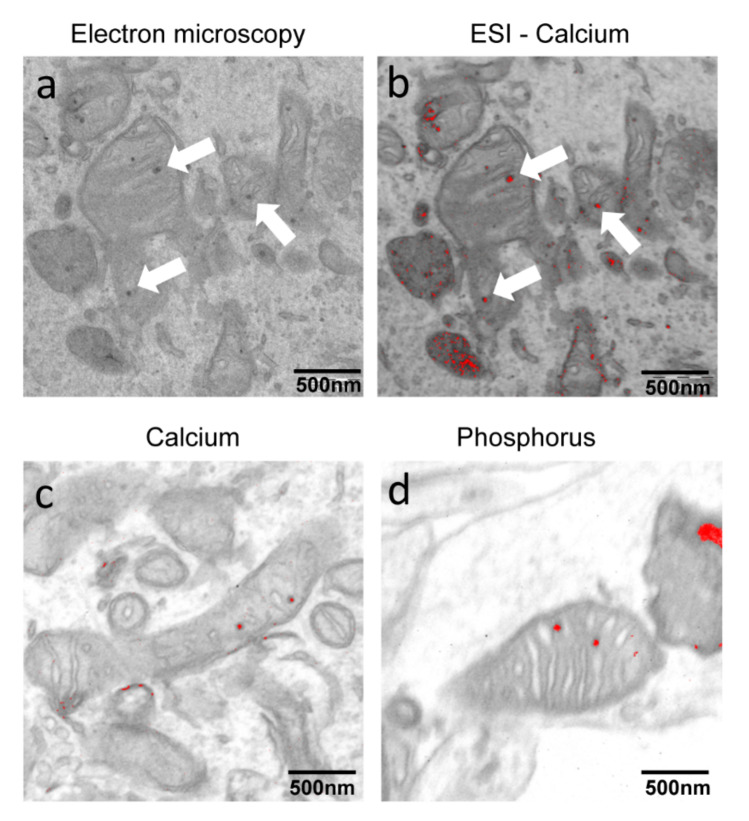
Electron spectroscopic imaging (ESI) analysis on PKAN iPS-derived neurons. (**a**) Representative images of ultrastructural analysis of mitochondria from fixed iPS-derived neurons examined with electron microscope. (**b**) Overlapping of the map obtained by ESI in which calcium is evidenced in red and of the same field visible in A. (**c**,**d**) Overlapping of the map obtained by ESI analysis in which calcium or phosphorus are respectively evidenced in red. Arrows point to electron dense granules present in mitochondria. Scale bar 500 nm.

The data confirmed those previously obtained [18] showing ~70% of Vglut1 positive neurons in all samples (Figure 1b) and indicating glutamatergic identity. A quantitative measurement of total iron content by ICP-MS revealed about two-fold increase of iron content in PKAN iPS-derived neurons with respect to the control neurons (Figure 1c). To verify if this larger amount of iron was detectable as deposits, we performed EM analysis showing the presence of electron dense dots, mainly located into mitochondria in PKAN patients (Figure 1d). To define the chemical composition of these dots, iPS-derived neurons, fixed in the absence of Ca^2+^ were analyzed by ESI. Unexpectedly, these analyses revealed that the dots contain calcium and phosphorus, thus establishing that they are composed of calcium phosphate (Figure 2).

Quantification of these dots, by counting their number in each mitochondria, showed that the percentage of mitochondria with dots was significantly higher in the iPS-derived neurons from all the PKAN patients analyzed, compared to controls (Figure 1e, left panel). Moreover, if we consider only mitochondria with at least three dots, the difference between PKAN patients and controls is highly significant (Figure 1e, right panel). To verify if the presence of these dots was specifically associated with the neuronal differentiation, we evaluated their presence also in fibroblasts and NPCs, and compared the data with control cells. The results, obtained by plotting the percentage of mitochondria with or without dots, indicated that mitochondrial electron dense dots increased significantly during differentiation only in PKAN iPS-derived neurons (Figure 1f).

### 2.2. Calpain1 Activity Increase in PKAN iPS-Derived Neurons

**Figure 3:**
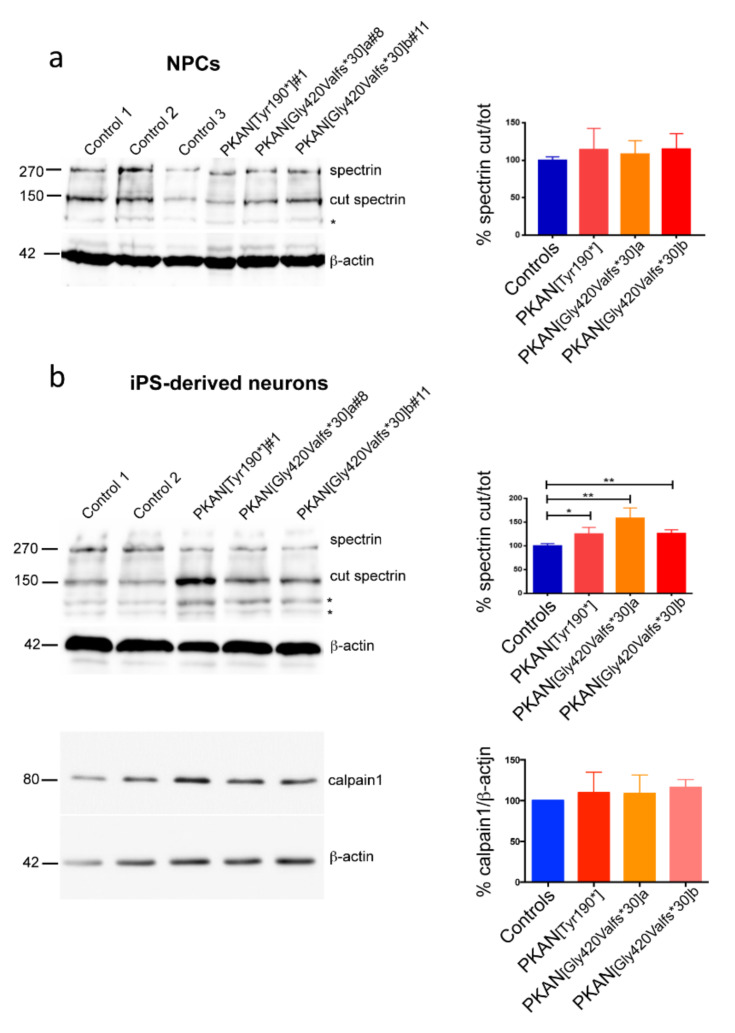
Analysis of the product (cut spectrin) of calcium-dependent calpain1 enzymatic activity. (**a**) Immunoblotting probed with antibody specific for spectrin on neuronal precursor cells (NPCs) and (**b**, top panel) on iPS derived-neurons (20 µg of total proteins). The asterisks indicate non-specific bands. (**b**, lower panel) Immunoblotting probed with antibody specific for calpain1 on iPS derived-neurons. β-actin was used as loading control in all the immunoblotting. Graph of the ratio of cut spectrin/total spectrin in neuronal precursor cells and in iPS derived-neurons and of calpain1/β-actin in iPS derived-neurons are shown on the right side of the respective blotting. The statistical analysis was conducted using student-*t*-test: * *p* < 0.05, ** *p* < 0.01. All data are presented as mean + SEM on at least three independent replicates.

The presence of calcium phosphate dots in mitochondria suggested an impairment of calcium homeostasis. This was verified by monitoring the activity of calpain1, a ubiquitous enzyme whose activity depends upon the calcium bound to its EF-hands motif. Calpain1 has great affinity for cytoskeletal proteins including the spectrin. Therefore, using immunoblotting we could obtain an estimation of the enzymatic activity by quantifying the bands related to cut and total spectrin, and calculating their ratio. Soluble cell extracts from cultures of NPCs and iPS-derived neurons differentiated for 21 days were probed with an antibody specific for the α-chain of the spectrin, able to recognize both the cut and the whole form. The results indicated that calpain1 showed similar activity in NPCs from PKAN patients and controls (Figure 3a). Conversely, the calpain1 activity was higher in PKAN iPS-derived neurons compared to controls (Figure 3b). This was not due to differences in calpain1 content, which was comparable in all the analyzed samples (Figure 3b). These data suggest a higher cytosolic Ca^2+^ content in PKAN iPS-derived neurons.

### 2.3. The Cytosol of PKAN iPS-Derived Neurons Shows Less Calcium Influx than Controls

**Figure 4:**
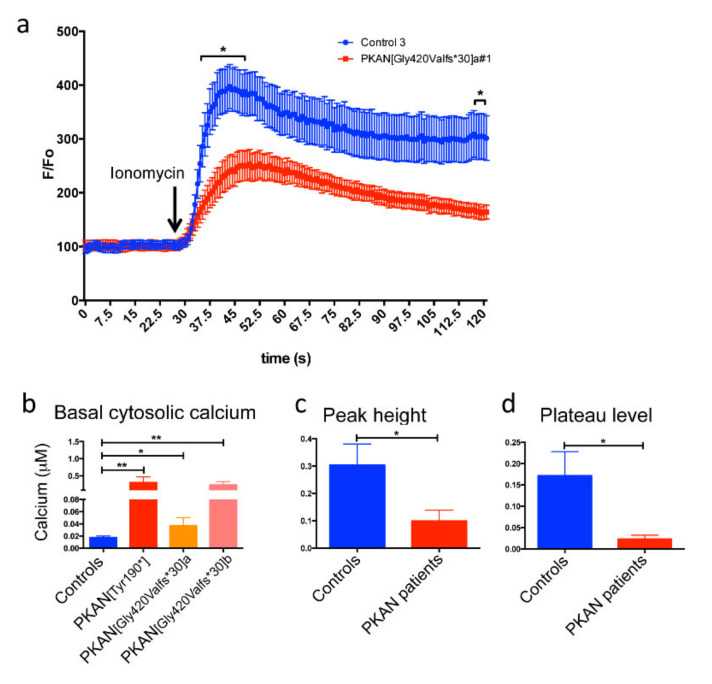
Fura Red qualitative/quantitative analysis of the cytosolic calcium content in iPS-derived neurons. (**a**) An example of graph of the analysis with the Fura Red AM probe in control and PKAN [Gly420Valfs*30]a iPS derived-neurons. (**b**) Graph showing the cytosolic calcium concentration in basal conditions obtained by measuring the fluorescence ratio of Fura Red following excitation at 405/488 nm and calculated on a calibration curve. (**c**) Graph showing the levels of cytosolic calcium, after addition of ionomycin, obtained by measuring the increase in fluorescence at the peak compared to baseline. (**d**) Graph showing the levels of cytosolic calcium, after addition of ionomycin, obtained by measuring the increase in fluorescence at the plateau compared to baseline. The statistical analysis was done using in a two-way ANOVA; in (**b**) one-way ANOVA; in (**c**) and (**d**) Student’s *t*-test: * *p* < 0.05, ** *p* < 0.01. All data are presented as mean + SEM on at least three independent replicates.

To confirm these data by a different approach, we performed calcium imaging that allows quantitative analysis of Ca^2+^ concentration. iPS-derived neurons differentiated for 21 days were loaded with 2 µM Fura Red AM, a ratiometric Ca^2+^ sensitive fluorescent dye, for 30 min at 37 °C. Then, the fluorescence emission in basal condition and after the addition of the Ca^2+^ ionophore ionomycin, was recorded for 2 min (Figure 4a). The extrapolation of quantitative data (see MM), obtained in basal conditions, indicated that Ca^2+^ concentration was higher in all the PKAN patients compared to control neurons (Figure 4b). As expected, after the addition of the ionomycin, the peak and the plateau fluorescence indicated that free cytosolic Ca^2+^ increased in all cells, but to different extents. The PKAN iPS-derived neurons showed an average lower increase in both peak and plateau fluorescence compared to controls (Figure 4c,d). We hypothesized that the higher cytosolic basal Ca^2+^ concentration impaired the Ca^2+^ equilibrium among different compartments.

**Figure 5:**
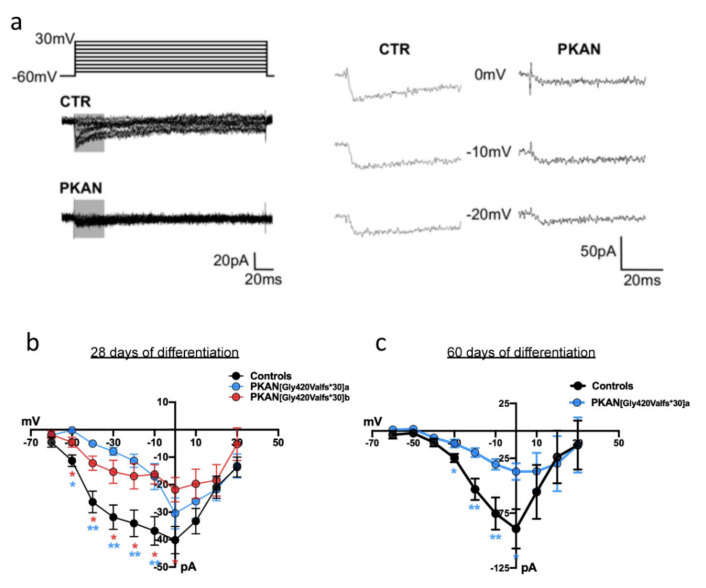
Voltage-dependent calcium currents recorded in iPS-derived neurons. (**a**) Example of calcium currents recorded in voltage-clamp at depolarizing levels from -60 to +30 mV in control (CTR) and PKAN iPS derived-neurons after 28 days from the start of differentiation. The right panel shows magnified portions of calcium currents included in gray boxes on the left. (**b**,**c**) Current-to-voltage plots from control and PKAN iPS derived-neurons 28 (panel **b**) and 60 (panel **c**) days after the start of the differentiation. Statistical analysis was performed using one-way ANOVA (* *p* < 0.05, ** *p* < 0.01). All data are presented as mean + SEM on at least three independent replicates (controls at 28 days = 40 cells; PKAN at 28 days = 14 cells. Controls at 60 days = 12 cells; PKAN at 60 days = 9 cells).

To further investigate cytosolic Ca^2+^ unbalance in iPS-derived neurons, we recorded voltage-gated Ca^2+^ currents at two different stages of neuronal maturation (28 and 60 days). Figure 5a shows examples of Ca^2+^ currents recorded from control and PKAN iPS-derived neurons. Current–voltage (IV) relationships are represented in Figure 5b,c. At early developmental stages (28 days) PKAN iPS-derived neurons showed smaller voltage-gated Ca^2+^ currents with respect to controls. One patient p.[Gly420Valfs*30]a was maintained longer (60 days), showing that this impairment became stronger. In agreement with the Fura Red data, these results support the hypothesis that cytosolic Ca^2+^ homeostasis is impaired in PKAN iPS-derived neurons.

### 2.4. Neuroimaging Confirms Calcium Accumulation In Vivo

**Figure 6:**
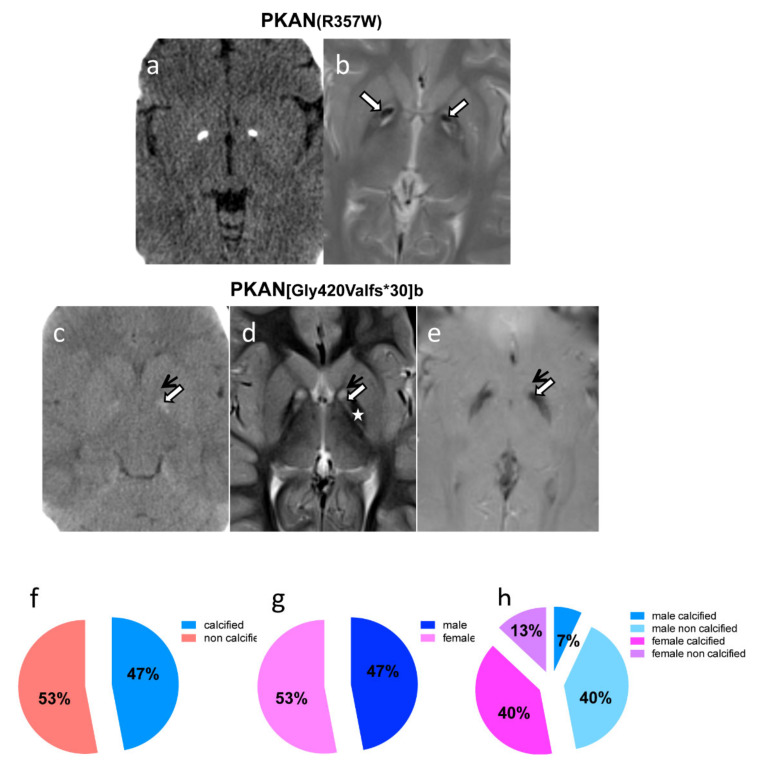
Imaging studies on in vivo PKAN patients’ brains. (**a**) Axial CT and (**b**) T2-w MR images show bilateral rock calcifications in the *globi pallidi*, corresponding to the strong hypointensity into the antero-medial portion hyperintensity of the eye-of-the-tiger sign (white arrows). (**c**) Axial CT, (**d**) T2-w and (**e**) GRE MR images, demonstrate that the slight calcifications (white arrows) lie into the antero-medial portion hyperintensity of the *globi pallidi* (black arrows), right in front of the back portion of the nuclei where there is iron deposition (star). (**f**–**h**) Graphical representation of the distribution of male and female and of calcified and non-calcified patients.

To correlate the data obtained in vitro on PKAN iPS-derived neurons we searched in the FINCB neuroradiological database to identify genetically defined PKAN patients, who underwent a CT scan of the brain. Over a total of 15 patients, whose CT scan was available, 47% showed either bilateral rock calcifications or slight calcifications of the globi pallidi (Table 1). All patients but one (HA185) displayed the ‘eye-of-the-tiger’ sign, the typical hallmark of PKAN, detected by MRI (Figure 6b,d,e); patient HA185 had basal ganglia atrophy, T2 hypointensity of the globus pallidus and a reticular aspect of the striati. In the ‘eye-of-the-tiger’ sign, T2-hypointensity of the back portion of the pallidi represents iron accumulation; T2-hypointensity into the antero-medial portion hyperintensity is due to calcifications that are well defined on brain CT (Figure 6). The reciprocal localization of iron and calcium in the globi pallidi showed in Figure 6 indicate that these two elements accumulate almost simultaneously and remain unchanged in subsequent follow-up.

A gender balance was present in our cohort (47% of patients are male and 53% are female) and despite the small number, we observed that a significant fraction of female patients presented calcium accumulation (75%, corresponding to 40% of total patients), while only a minor fraction of male (14%, corresponding to 7% of the total patients) showed calcifications (Table 1 and Figure 6). We did not notice any correlation between calcium accumulation and patients’ age, disease duration or type of mutation. Interestingly, we observed the convergent finding of calcium accumulation in iPS-derived neurons and in the brain of patient HA101 (Table 1 and Figure 6). However, in her older brother, patient HA102 (Table 1), we detected calcium granules in iPS-derived neurons, but his CT scan showed no calcifications. In these two subjects, CT scan was performed in two consecutive days, when patient HA101 was 5 years old and patient HA102 was 8 years old. On the same days, a skin biopsy to obtain fibroblasts was carried out. As for the other patient, from whom iPS-derived neurons were obtained and showed calcium granules, a CT scan was not available and could not be performed.

## 3. Discussion

In this work, we present a new phenotype detectable in PKAN iPS-derived neurons. Despite the evidence of higher amount of total cellular iron content, these neuronal cells showed the presence of mitochondrial aggregates composed by calcium phosphate precipitates. In particular, by analyzing the cells at different stages of differentiation, we noticed that the percentage of mitochondria with aggregates increased in PKAN iPS-derived neurons, compared to control cells, while they were similar to the control in fibroblasts and neuronal precursors. This data indicated that the mitochondrial aggregates increased during late stage of neuronal differentiation. Moreover, the increased activity of calpain1, a calcium-dependent protease in the cell, detected in iPS-derived neurons, was not observed in neuronal precursors, confirming that the alteration of calcium homeostasis was specific to the neuronal cells of PKAN patients. Data derived from calcium imaging show that, under basal conditions, a greater amount of calcium is present in PKAN iPS-derived neurons cytosol as compared to controls. This feature may have an effect on the activity of calcium channels [21], or may cause a drop in the driving force for this ion, thus reducing Ca^2+^ influx into the cytosol. These hypotheses are supported by both calcium-imaging results, which revealed a lower fluorescence increase upon ionomycin stimulation, and by electrophysiology recordings, in which lower peak Ca_v_ currents were recorded in iPS from PKAN patients with respect to control. Although we cannot identify the origin of calcium leakage toward the cytosol, based on previously reported data [22,23,24,25,26], we can speculate that an altered iron metabolism may cause RyR-mediated Ca^2+^ leakage from the ER, and/or disrupt the mitochondrial calcium buffer ability also in PKAN neurons.

These data prompted us to investigate if not only in vitro but also in PKAN patients in vivo, calcium accumulation was detectable. Recently, a case report showed basal ganglia calcifications in a patient affected by PKAN [27]. A survey of a cohort of 15 genetically defined PKAN patients who underwent CT scan demonstrated calcium accumulation in 47% of the cases. These patients were all clinically homogeneous, and no correlation between genotype and phenotype could be established, nevertheless, we noticed a significant prevalence of calcification in female versus male patients.

Previous work indicated the presence of estrogen binding sites, different from the nuclear estrogen receptor, in the brain. Specifically, in the rat brain, it was reported that estradiol binds to a subunit of ATP synthase, the complex V of the mitochondrial respiratory chain, thus modulating energy production [28]. Moreover, it is known that estrogen may modulate L-type calcium channel by a physical interaction, which leads to modifications of the intracellular calcium levels, thus playing a crucial role in the central nervous system in many neurodegenerative diseases [29].

However, we still do not know if there is any correlation between calcium deposits and hormonal status, or other genetics or epigenetics factors, but it is interesting to note that both siblings HA101 (female) and HA102 (male) showed the presence of calcium accumulation in iPS-derived neurons, while their CT scans revealed the presence of bilateral calcification only in the female patient.

Our knowledge on the molecular mechanisms linking iron with calcium accumulation in this disorder is limited. It is known that iron and calcium are essential ions for the maintenance of neuronal function, but their reciprocal homeostasis should be finely tuned to avoid deleterious effects. Excessive iron causes oxidative stress leading to modification of crucial proteins involved in calcium homeostasis [30]. In turn, an increase of calcium levels causes mitochondrial dysfunction and loss of iron homeostasis, thus suggesting that their optimal relationship is crucial to assure a healthy neuronal activity.

Any perturbation of this equilibrium can cause damage to neuronal cells resulting in neuronal degeneration. In fact, various pathological phenomena leading to an altered signaling of intracellular calcium can activate cell death pathways [31].

A harmful iron-calcium connection was already reported in several neurodegenerative disorders, such as Alzheimer and Parkinson diseases and ALS [30]. Notably, another group of genetic diseases, globally referred to as Primary Familial Brain Calcification (PFBC), is characterized by calcium phosphate deposition in basal ganglia and other brain regions, leading to progressive neurodegeneration presenting with movement disorders, cognitive decline and psychiatric disturbances [32]. Here, we report for the first time in iPS-derived neurons, that calcium accumulation is also a consistent finding in neurodegeneration associated with panthothenate kinase deficiency, a disorder primarily characterized by iron overload.

The most reliable current hypothesis regarding PKAN pathogenic mechanisms correlates PANK2 mutations to an altered mitochondrial CoA production that may cause a series of metabolic defects associated with lipid metabolism necessary for membrane remodeling. These alterations can damage the membranes of mitochondria and alter their functions [18,33] suggesting the possibility that lipid dys-homeostasis may play an important role in the pathogenesis of the disease. This hypothesis is also supported by recent data indicating reduced TfR1 palmitoylation in PKAN and other NBIA patients’ fibroblasts, resulting in an abnormal TfR1 recycling, which could promote iron incorporation [34]. We also demonstrated that PKAN iPS-derived neurons showed altered iron metabolism but not iron deposition, causing an increase in ROS production and an alteration of mitochondrial membrane potential [18]. This, in turn, can cause an increase in calcium levels, which can further damage the mitochondria, disrupting synaptic functioning and reducing neuronal survival. Remarkably, we observed that in patients who underwent CT scan before or at the same time of MRI, calcium accumulation was already present. Despite the limited number of patients, this observation suggests that calcium accumulation probably occurs simultaneously to iron accumulation. This would be in agreement with the data on PKAN iPS-derived neurons, demonstrating impairment of iron metabolism as a first step of the pathogenic process. This could then drive calcium accumulation, which we observed in neurons in a specific temporal window, but not iron accumulation, a process probably occurring at later stages of disease, and not detected in neurons under the period of our observations. However, we cannot exclude that other factors, like the homogeneity of cellular composition or the specificity of our neuronal model, could influence the sequence of the detrimental facts.

Nevertheless, the connection between impaired CoA synthesis, a cofactor of fundamental importance for the life of each cell, and calcium and iron accumulation limited to the central nervous system and, more precisely, to the *globus pallidus*, remains unclear.

Here, we demonstrated that intracellular calcium accumulation in iPS-derived neurons generated from PKAN patients correlates with the presence of brain calcifications detected by CT scan. Limitations due to sensitivity of the in vivo detection technique, to disease duration and genetics or hormonal factors are recognized. However, to the best of our knowledge, there are no differences in the clinical presentation or prognosis of the disease in the different patients, which could be ascribed to the presence/absence of calcium.

## 4. Materials and Methods

### 4.1. Patients

We reviewed the clinical, radiological and genetic data of a cohort of 15 genetically defined PKAN patients (Table 1) assessed at the Fondazione IRCCS Istituto Neurologico “Carlo Besta”, Milan, Italy, between January 2000 and December 2019. The patients came from different areas of Italy and eight were female. The mean age at clinical onset was 8 years (range 1–24 years). The study was performed as per approval of ethics committee of the Fondazione Istituto Neurologico C. Besta, and in agreement with the Declaration of Helsinki principles. All the patients involved gave their written informed consent, approved by the same ethics committee, with codes CI 66a/b for brain MRI (date 19-2-2019) and CI 28a/b for CT scan (date 11-11-2015) respectively.

### 4.2. Fibroblasts Culture and iPSC Generation

Fibroblasts from healthy subjects, one newborn male and two females, one newborn and one adult (27 years of age) were purchased from ATCC, while fibroblasts from PKAN patients were obtained from the Movement Disorders Bio-Bank available at the Medical Genetics and Neurogenetics Unit of the Fondazione IRCCS Istituto Neurologico “Carlo Besta”. One female patient, 12 years old at time of skin biopsy, was a carrier of the c.[569_570insA] homozygous mutation that caused the introduction of a p.[Tyr190*] premature stop codon, while two siblings, a male and a female (8 years old and 5 years old at time of skin biopsy, respectively), carried the same c.[1259delG] homozygous mutation that caused a frameshift p.[Gly420Valfs*30] mutation. The reference sequence used for the PANK2 mutations was NM_153638. These patients were previously published with the old nomenclature, p.Y190X and p.F419fsX472, respectively [18,33]. All subjects gave their written consent for the skin biopsy procedure and for the use of the sample material for research purposes. The iPSC generation was previously described in [18].

### 4.3. Generation of Human Neuronal Precursors Cells and iPS-Derived Neurons

Neuronal precursor cells (NPCs) were obtained from iPSC with the following procedure: embryo bodies (EBs) were formed by dissociation of iPSC colonies with passaging solution (Miltenyi Biotec, Bologna, Italy) and plating onto low-adherence dishes in mTeSR medium supplemented with Noggin (0.5 mg/mL, R&D System, Minneapolis, MN, USA), SB431542 (5 mM, Sigma-Aldrich, Milano, Italy), N2 (1:200, Life Technologies, Milano, Italy), and 1% P/S for 10 days. To obtain rosettes, EBs were plated onto Matrigel Matrix (Corning, Torino, Italy)-coated dishes in DMEM/F12 (Sigma-Aldrich) with N2 (1:200), 1% NEAA (Life Technologies) and 1% P/S. After 10 days, rosettes were dissociated with Accutase and re-plated onto Matrigel-coated dishes containing NPC medium (DMEM/F12, N2 (1:200), B27 (1:100), 1% P/S and FGF2 (20 ng/mL)). Homogeneous populations of NPCs were achieved after 3–5 passages with Accutase in the same conditions.

The iPS-derived neurons were obtained as previously described [18]. Briefly, NPCs were transduced with a lentivirus (LV) expressing Ngn2 cDNA under the control of a tetracycline responsive promoter and an LV expressing rtTA. LV was produced as previously described [35]. NPCs were seeded on Matrigel-coated wells and differentiated in medium containing Neurobasal (Life Technologies), BDNF (10 ng/mL, Peprotech, London, UK), NT-3 (10 ng/mL, Peprotech), B27, 1% P/S, and doxycycline (2 µg/mL, Sigma). One clone for each healthy subject (control 1, 2, 3), one from patient p.[Tyr190*] (#1) and three from patients p.[Gly420Valfs*30]a (#1, 3, 8) and b (#2, 5, 11) were used in this work.

### 4.4. Immunofluorescence

A total of 4 × 10^4^ NPCs were seeded on Matrigel-coated covers and differentiated to glutamatergic neurons. Cells were fixed in 4% paraformaldehyde and processed as previously described [18]. Immunofluorescence was performed using specific antibodies: rabbit anti microtubule-associated protein 2, Map2 (1:400; AB5622, Sigma-Aldrich); guinea pig anti vesicular glutamate transporter 1, Vglut1 (1:200; 135304, Synaptic System, Goettingen, Germany); Alexa fluor 488 donkey anti rabbit IgG (1:800; IS20015, Immunological Sciences, Roma, Italy); Alexa fluor 594 goat anti guinea pig IgG (1:800; A11076, ThermoFisher Scientific, Monza, Italy). Images were acquired by fluorescence microscope Zeiss Axio Observer.Z1 equipped with Hamamatsu EM-CCD 9100-02 camera and Volocity acquisition software.

### 4.5. Immunoblotting

A total of 3 × 10^5^ NPCs were seeded on 6 well Matrigel-coated plates and differentiated to neurons. Soluble cellular extracts for immunoblotting were obtained by lysing cells in 20 mM Tris–HCl, pH 7.4, 1% Triton X-100, and a protease inhibitor cocktail (Roche, Monza, Italy) followed by centrifugation at 16,000 g for 10 min. Soluble proteins (25 µg) were separated by sodium dodecyl sulfate–12% poly-acrylamide gel electrophoresis (SDS-PAGE), and immunoblotting was performed using specific antibodies: mouse anti spectrin α-chain (1:1000; MAB1622, Merck-Millipore, Milano, Italy); mouse anti calpain1 (1:1000; MA1-1679, ThermoFisher Scientific); mouse anti β-actin (1:6000; A5441, Sigma-Aldrich) followed by peroxidase-labeled secondary antibodies (Sigma-Aldrich). The signal was then revealed using the ECL-chemiluminescence kit (GE Healthcare, Milano, Italy) and detected with ChemiDoc MP Imaging System (BIORAD, Segrate, Italy). Total protein contents were measured using the BCA protein assay calibrated with bovine serum albumin (ThermoFisher Scientific).

### 4.6. Electron Microscopy (EM) and Electron Spectroscopic Imaging (ESI)

The iPS-derived neurons were fixed in 4% paraformaldehyde and 2.5% glutaraldehyde, post-fixed with 2% OsO4, washed, dehydrated and embedded in Epon812. Thin sections were stained with uranyl acetate and lead citrate and examined in a EFTEM Leo912 electron microscope (Zeiss, Milano, Italy). Images were randomly obtained in blind conditions to the examiner.

Specimens were fixed as previously described for EM with the omission of Ca^2+^ in the buffer. Ultrathin sections were subjected to ESI analysis. The images, acquired by the EFTEM Leo912 electron microscope, were first examined at 250 eV (i.e., at an energy loss where scattered electrons of most elements contribute to the image), to provide a general view of the ultrastructural organization. The patterns of net calcium distribution were then obtained by computer-assisted processing of two images collected below (320 eV and 330 eV) and one beyond the calcium L_2,3_ absorption edge at 350eV. Similarly, for phosphorus distribution by two images collected below (100 eV and 120 eV) and one beyond the phosphorus L_2,3_ absorption edge at 153 eV. The obtained map, represented in pseudo-colours, was superimposed to the corresponding 250 eV image.

### 4.7. Ca^2+^ Imaging

We measured Ca^2+^ imaging by the ratiometric dye Fura Red AM (Invitrogen, Carlsbad, CA, USA). NPCs (5 × 10^4^ cells) were seeded on Matrigel-coated cover glasses and differentiated to glutamatergic neurons as described above. After 21–28 days, they were incubated for 30 min at 37 °C with 2 µM Fura Red AM (Molecular Probes, Eugene, OR, USA) and 0.01% pluronic acid (Sigma-Aldrich) in HBSS (137 mM NaCl, 5.4 mM KCl, 2 mM CaCl_2_, 0.5 mM MgCl_2_, 0.4 mM KH_2_PO_4_, 0.4 mM MgSO_4_, 0.3 mM Na_2_HPO_4_, 25 mM Hepes pH 7.35) containing 10 mM glucose. Cells were then washed and maintained in HBSS with 10 mM glucose and images were obtained with UltraVIEW ERS Spinning Disk Confocal Microscope (PerkinElmer, Milano, Italy). The samples were excited at two wavelengths sequentially, 405 nm and 488 nm, and the images collected by EM-CCD Hamamatsu C9100 camera at emission higher than 600 nm; acquired images were then managed using Volocity software. First the fluorescence emission was measured in resting conditions, then, the iPS-derived neurons were stimulated with Ca^2+^ ionophore ionomycin (1 µM) (Sigma-Aldrich). By calculating the ratio of the fluorescence emitted following the excitation at the two wavelengths, it is possible to estimate the amount of free Ca^2+^ present in the cytosol in basal conditions (the greater the ratio, the greater the Ca^2+^ concentration), calculated on a calibration curve. Both the peak and the plateau fluorescence were measured as the difference with respect to the basal level.

### 4.8. Electrophysiology

Voltage-gated Ca^2+^ currents on iPS-derived neurons were recorded after 28 and 60 days of differentiation. Cells were continuously perfused with artificial cerebrospinal fluid (ACSF) containing the following: 105 mM NaCl, 2.5 mM KCl, 25 mM NaHCO_3_, 2 mM CaCl_2_, 1 mM MgCl_2_, 1.25 mM NaH_2_PO_4_, 11 mM glucose, 20 mM TEA, 4 mM 4AP, 0.001 mM TTX, (bubbled with 95% O_2_ and 5% CO_2_, pH 7.3 at RT). Whole-cell pipettes were filled with the following solution: 137 mM CsCl, 4 mM MgCl_2_, 10 mM EGTA, 10 mM HEPES, 4 mM Na_2_-ATP, 0.3 mM Na_2_-GTP (pH adjusted to 7.3 with CsOH, tip resistance 5–6 MΩ). Ca^2+^ currents were recorded in voltage clamp mode by clamping membrane voltage from −60 up to 30 mV by 10 mV steps (200 ms). Traces were recorded using a digital amplifier (Multiclamp 700B, Molecular Devices, San Jose, CA, USA) interfaced with a PC through an analog-to-digital board (Digidata 1440A, Molecular Devices). Signals were acquired and analyzed using pClamp software (Molecular Devices). All reagents were supplied by Sigma-Aldrich.

### 4.9. Inductively Coupled Plasma Mass Spectrometry (ICP-MS)

Cell homogenates were digested in 70% nitric acid and deionized water (1:1) for 1 h at 70 °C. The digested samples (0.1 mL) were diluted with deionized water (2.9 mL). Iron concentration was then determined by ICP-MS on an ELAN DRC II (PerkinElmer) using the analytical technique total quant (begin mass 49 a.m.u., end mass 58 a.m.u.) with external calibration and using the DRC with ammonia (flow 0.7 mL/min, RPq 0.65). The instrument was calibrated using a standard solution (Multielement ICP-MS Calibration Standard 3, Matrix per Volume: 5% HNO_3_ per 100 mL, Perkin Elmer Plus) at a concentration of 10 μg/L. Each sample was analyzed thrice. The method accuracy was determined in natural water reference materials (NIST 1643F, National Institute of Standard and Technology, Gaithersburg, MD). The coefficients of variation (CV) ranged from 4% to 8% among series and from 6% to 12% between series. The limit of detection (LOD), calculated as 3 standard deviations of the background signal obtained on 10 white samples, was 0.0006 μg/L.

### 4.10. Brain MRI and CT Scan

All 15 patients underwent one or more brain CT and one or more conventional brain MR imaging studies on a 1.5 Tesla Avanto scanner (Siemens Medical Solutions, Erlangen, Germany) or a 3 Tesla Achieva scanner (Philips Medical Systems, Eindhoven, The Netherlands). A trained neuroradiologist (L.C., with 26 years of experience) reviewed all MRI scans.

### 4.11. Statistical Analysis

Statistical methods were not employed to predetermine sample size in the experiments. All the experiments were performed at least in triplicate; data were analyzed using GraphPad Prism. In general, for normally distributed data, two-tailed unpaired Student’s *t*-test and one- or two-way ANOVA followed by Bonferroni post-test were used. For non-normally distributed data, Kruskal-Wallis test was used. The data are reported as the mean + SEM. The *p*-value < 0.05 was considered statistically significant.

## 5. Conclusions

By employing the iPS-derived neurons from PKAN patients the current study highlights that, in a disorder primarily characterized only by iron overload, calcium accumulation is a consistent finding in neurodegeneration associated with panthothenate kinase 2 deficiency. Interestingly, this phenotype was confirmed also in basal ganglia of patients’ brains. The complex interplay between iron and calcium must be tightly regulated in order to maintain correct cell functionality. Although further studies are necessary to clarify the pathogenic mechanism, this work suggests that calcium accumulation, probably occurring simultaneously to iron accumulation, must be considered as an additional player in PKAN.

## Figures and Tables

**Table 1:** PKAN patients subjected to CT scan.

Patient’s Code (Sex)	PANK2 Mutations ^$^ (cDNA)	PANK2 Mutations ^$^ (Protein)	Date of CT Scan	Globi Pallida Calcifications	DBS	Date of Brain MRI	Age of Disease Onset
mt4245 (f)	c.[821_822delCT]; [1561G > A]	p.[Leu275Valfs*16]; [Gly521Arg]	2014	bilateral rock calcifications	DBS	2006; 2013	2 years
HA44 (f)	c.[1069C > T]; [1069C > T]	p.[Arg35Trp]; [Arg35Trp]	2013; 2015	bilateral rock calcifications	DBS	2008; 2009	24 years
HA143 (f)	c.[965A > G]; [1561G > A]	p.[Glu322Gly]; [Gly521Arg]	2015	bilateral rock calcifications	DBS	2006; 2015	16 years
HA31 (f)	c.[821_822delCT]; [del ex 1-4]	p.[Leu275Valfs*16]; [not translated]	2000	bilateral rock calcifications		2001; 2002	1 year
HA26 (m)	c.[1441C > T]; c.[966G > T]	p.[Arg481*]; p.[Glu322Asp]	2017	no calcifications		2014; 2015; 2017	17 years
HA35 (m)	c.[790C > T]; [790C > T]	p.[Arg264Trp]; [Arg264Trp]	2015	bilateral calcifications	NA	2008; 2009	NA
HA101 (f)	c.[1259delG]; [1259delG]	p.[Gly420Valfs*30]; [Gly420Valfs*30]	2007	bilateral calcifications		2007; 2008; 2009	4 years
HA102 (m)	c.[1259delG]; [1259delG]	p.[Gly420Valfs*30]; [Gly420Valfs*30]	2007	no calcifications		2007; 2008; 2009; 2013	7 years
HA134 (m)	c.[1499A > T]; [621-1G > A]	p.[Asn500Ile]; ?	2008	no calcifications		2004; 2008	2 years
mt4597 (m)	c.[683T > C]; [683T > C]	p.[Phe228Ser]; [Phe228Ser]	2013	no calcifications	DBS	2006; 2008; 2011	2 years
HA167 (m)	c.[821_822delCT]; [965A > G]	p.[Leu275Valfs*16]; [Glu322Gly]	2011	no calcifications		2009; 2010; 2011	9 years
HA316 (f)	c.[821_822delCT]; [1441C > T]	p.[Leu275Valfs*16]; [Arg481*]	2015	bilateral slight calcifications		2005 (normal); 2007 (pathological iron); 2013	18 months
HA185 (f)	c.[36T > A]; [137A > T]	p.[His12Gln]; [Asp46Val]	2010	no calcifications		2010; 2011; 2012; 2015; 2019	12 years
BDM862 (f)	c.[1236-1G > A]; [1561G > A]	p.?;[Gly521Arg]	2015; 2017	no calcifications		2015; 2016	5 years
LDM709 (m)	c.[982-1G > A]; [1561G > A]	p.?;[Gly521Arg]	2019	no calcifications		2017; 2019	8 years

Patients identified by code, sex and mutations. Calcification, deep brain stimulation (DBS), date of CT scan and brain MRI, and age of onset are indicated. ^$^ Following the HGVS-nomenclature [20], PANK2 reference sequence NM_153638. ? = unknown effect of the mutation on the protein.

## Abbreviations

CoA	Coenzyme A
ICP-MS	Inductively coupled plasma mass spectrometry
iPSC	Induced Pluripotent Stem Cells
NBIA	Neurodegeneration with Brain Iron Accumulation
PANK2	pantothenate kinase 2
PKAN	Pantothenate Kinase-Associated Neurodegeneration
